# PCA of Running Biomechanics after 5 km between Novice and Experienced Runners

**DOI:** 10.3390/bioengineering10070876

**Published:** 2023-07-24

**Authors:** Xinyan Jiang, Datao Xu, Yufei Fang, István Bíró, Julien S. Baker, Yaodong Gu

**Affiliations:** 1 Research Academy of Medicine Combining Sports, Ningbo No. 2 Hospital, Ningbo 315010, China; 2 Faculty of Sports Science, Ningbo University, Ningbo 315211, China; 3 Doctoral School on Safety and Security Sciences, Obuda University, 1034 Budapest, Hungary; 4 Faculty of Engineering, University of Szeged, 6720 Szeged, Hungary; 5 Faculty of Engineering, University of Pannonia, 8201 Veszprém, Hungary; 6 Department of Sport, Physical Education and Health, Hong Kong Baptist University, Hong Kong, China; 7 Department of Radiology, Ningbo No. 2 Hospital, Ningbo 315010, China

**Keywords:** principal component analysis, running experience, running-related injuries, biomechanics

## Abstract

Increased running experience appears to lower the risk of running-related injuries, but the mechanisms underlying this are unknown. Studying the biomechanics of runners with different running experiences before and after long-distance running can improve our understanding of the relationship between faulty running mechanics and injury. The purpose of the present study was to investigate if there were any differences in lower-limb biomechanics between runners after a 5 km run. Biomechanical data were collected from 15 novice and 15 experienced runners. Principal component analysis (PCA) with single-component reconstruction was used to identify variations in running biomechanics across the gait waveforms. A two-way repeated-measures ANOVA was conducted to explore the effects of runner and a 5 km run. Significant runner group differences were found for the kinematics and kinetics of lower-limb joints and ground reaction force (GRF) with respect to the magnitude across the stance phase. We found that novice runners exhibited greater changes in joint angles, joint moments, and GRFs than experienced runners regardless of the prolonged running session, and those patterns may relate to lower-limb injuries. The results of this study suggest that the PCA approach can provide unique insight into running biomechanics and injury mechanisms. The findings from the study could potentially guide training program developments and injury prevention protocols for runners with different running experiences.

## 1. Introduction

Running is one of the most common sports worldwide because of the benefits it brings to runners. Each step of running can result in minor load changes; however, the accumulation of repetitive loads can lead to lower-limb cumulative overload, which is associated with overuse injuries [1,2,3]. A recent study reported the rate of running-related injuries (RRIs) as high as 85% [4]. At the 12 week follow-up stage, about 30% of runners had lower-limb injuries [5], and these injuries often have long-term effects [6]. Given the popularity of running and the high RRI risks, efforts are needed to identify injury mechanisms and prevention strategies.

Notably, it appears that lacking running experience is associated with a higher risk of RRI. Runners with years of running experience may have more adapted musculoskeletal systems, while novice runners may not have the same tolerance for running loads [7]. Novice runners are particularly vulnerable to injury in all runner groups. Studies [7,8,9] have shown that their injury rate was higher than that of experienced runners; the rate of injury risks was 17.8 per 1000 h of running against 7.7, respectively. Placing an emphasis on RRI prevention for novice runners is essential, as early injuries can be a barrier to continuing the running program [10]. Running biomechanics are increasingly considered important factors in the study of injury development. Boyer et al. [11] found differences in pelvic rotation, hip internal rotation, and hip and knee abduction and adduction angles during running between lower and higher mileage runners. Quan et al. [12] found that, compared with experienced runners, runners with less experience had a greater plantar flexion angle, dorsiflexion angle, range of motion (ROM), plantar flexion moment, and angular velocity in the ankle joint, and a greater flexion angle and range of motion in the hip joint, which indicate higher injury risks. A greater peak hip internal rotation angle was found among novice runners, which may be linked to knee injuries [10]. Experienced runners also showed less variability in stride interval than novice runners, which indicated that larger running volumes could develop stable and consistent movement patterns [13]. However, Agresta et al. [14] suggested that running experience does not change joint kinematics and kinetics or ground reaction force (GRF) variables during running. They suggested that the importance of expertise in preventing injury may not lie in enhanced running mechanics, but rather in enhanced motor patterns and functional adaptation to the environment or biological stresses.

All sustained physical activities produce varying levels of fatigue in the body. This is especially obvious in running, as running is one of the most popular exercises. The stress, shear, strain, and impact forces increase while exercising in a fatigued state [15,16]. Studies [17,18,19] have suggested that fatigue can induce alterations in running biomechanics after prolonged running. Some studies [19,20,21] on long-distance running biomechanical changes focused on recreational runners. Experienced runners may be less susceptible to fatigue than the novice group because of their greater training status. Strohrmann et al. [22] found that the trunk forward lean increased and the heel lift decreased with running exhaustion among runners of various skill levels, with beginner runners having more noticeable changes. However, the group of expert runners only included three subjects, and the other groups only included six subjects. Maas et al. [23] concluded that novice runners have larger peak forward trunk lean and hip abduction angle than competitive long-distance runners when exhausted. This study only investigated the kinematic parameters of running, but not the kinetic parameters. The extent to which runners with different running experiences also differed in their biomechanical responses to prolonged running has not been fully substantiated in the literature. Previous research on the effects of experience on running biomechanics has not been consistent, and there have been limited studies focusing on both kinematic and kinetic differences of long-distance running on runners with different experience levels. Thus, this study conducts a comprehensive analysis of the lower-limb biomechanics on novice and experienced runners with a prolonged running session.

Principal component analysis (PCA) is a method based on a mathematical algorithm that reduces data dimensionality while retaining the majority of the common modes of variation in the dataset and providing information that may significantly increase classification accuracy. It is used to explain a set of correlated variables [24,25,26]. PCA has been widely utilized to investigate human movement tasks such as running [11,24], lifting [27], and walking [28]. The PCs primarily maintained information regarding magnitude, difference, and phase shift [29]. Mantovani et al. [30] used PCA to compare the gait adaptations during walking after different hip arthroplasty surgical approaches and demonstrated that PCA could identify significant pattern delays in a certain group, whereas previous studies based on discrete parameters were unable to do so. O’Connor et al. [31] used PCA to evaluate the entire waveform of the cutting task and discovered that PCA revealed gender differences in cutting. The advantages of the PCA method are that it permits a more comprehensive way of evaluating motion modes and has the potential to be a meaningful discriminator of sports-related injury risk [31,32].

PCA can reduce locomotion and time series data without losing temporal information, and it produces independent principal components and scores [33]. By analyzing the modes of variation via PCA during running, it is possible to explain specific patterns in a set of variables. Therefore, through PCA analysis, differences between groups in joint motion of all lower-limb joints and GRF can be investigated systematically. If the running experience of runners and prolonged running sessions are risk factors for developing running-related injuries, then differences in lower-limb biomechanics may be expected. A distance of 5 km is widely regarded as attainable for most individuals, even for novice runners with regular exercise habits. It is unlikely to cause harm but still provides sufficient distance to elicit adaptations in running performance. The purpose of the present study was to explore the effect of a 5 km run between novice and experienced runners via PCA. We hypothesized that (1) running biomechanics would differ between novice runners and experienced runners in a prolonged running session, and (2) joint angles, joint moments, and ground reaction forces (GRFs) based on PCA would be more sensitive and effective in identifying group differences.

## 2. Materials and Methods

### 2.1. Participants

The required sample size was determined on the basis of a previous study [23]. A sample size of 12 runners per group was calculated, considering an effect size of 0.93 and a power of 0.9. In order to accommodate any potential data loss, 30 healthy male runners were enrolled in this study, including 15 experienced runners and 15 novice runners (Table 1). Novice runners were defined as those who ran 2–10 km per week and did not take part in a running competition or training program. However, it is important to note that they did have regular exercise habits and reported a minimum score of 5 out of 10 on the Tegner activity scale. Experienced runners consistently run at least 30 km per week and have more than 3 years of running experience [23,34]. Participants were heel-strike runners, with their dominant leg being the right (which was defined as the leg that was preferred for kicking a ball). Both novice and experienced runners had treadmill running experience and had no lower-limb injuries or musculoskeletal system disorders in the 6 months before the test. This study was a cross-sectional controlled study. The Institutional Review Board of Ningbo University approved each participant’s written informed consent prior to the study.

### 2.2. Experimental Procedures

**Figure 1:**
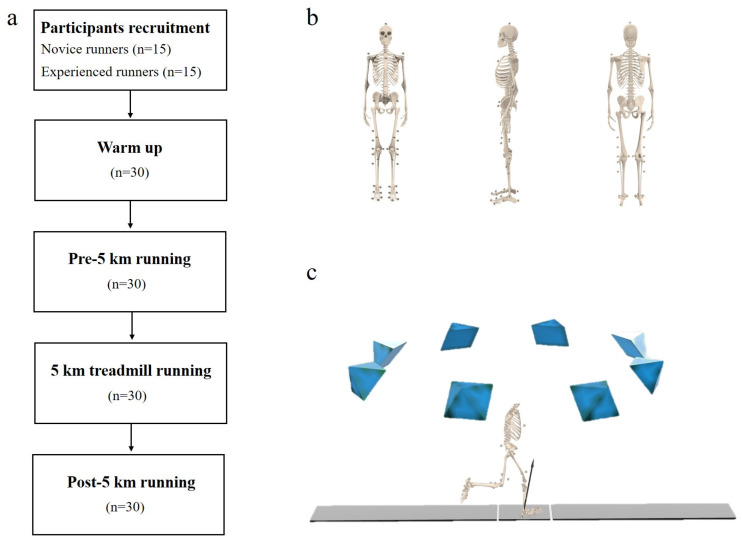
Illustration of testing protocol: (**a**) flowchart of participation in this study; (**b**) marker placement; (**c**) kinematic and kinetic data collection.

All runners wore tight pants and uniform running shoes (ART NO.11725599-7, ANTA) provided by the researchers, which minimized the influence of footwear on running biomechanics. Runners were given sufficient time to adjust to the running shoes. Thirty-nine retroreflective markers were located on participants. Markers were used to identify the trunk, hip, knee, and ankle segments; this model has been validated by previous research [19]. An eight-camera Vicon motion capture system (Vicon Metrics Ltd., Oxford, UK) and a force plate (AMTI, Watertown, MA, USA) were used to record running biomechanical data. Kinematics and kinetics data were recorded at 200 Hz and 1000 Hz, respectively. The testing protocol is presented in Figure 1. The participants were given 10 min to get warmed up and acquainted with the laboratory and testing procedures before the test started. Data for baseline running (pre-5 km running) were gathered at their preferred running speed; this speed was defined as a “natural running pace” [35]. The self-selected running speed was used for all data collection (novice runners were recorded at 3.28 ± 0.30 m/s, while experienced runners were recorded at 3.39 ± 0.32 m/s). The running speeds in the tests were monitored and controlled by two timing gates. To successfully collect running trials, runners had to maintain their gait while striking the ground force plate with their right foot only.

Each participant completed three successful running trials of pre-5 km running. For each trial, runners ran toward the ground force plate from 7 m away and continued to run for 5 m past the ground force plate. The ground runway consisted of a hard concrete surface, which differs from the surface of a treadmill. After completing the baseline running trials, runners ran 5 km at their preferred running speeds on the treadmill (Satun h/p/cosmos, Nussdorf-Traunstein, Nußdorf, Germany) with the slope of the treadmill set at 0°. The heart rate of each runner was continuously monitored during the treadmill run (RS 400; Polar Electro Oy, Kempele, Finland), and the Borg Scale was used to rate perceived exertion. The purpose of heart rate detection in the experiment was to protect runners from potential dangers if their heart rates exceeded the maximum. The Borg Scale was primarily used to assess the runners’ level of fatigue throughout the 5 km run in order to avoid the potential problems that runners may encounter when they are too exhausted to continue. Participants performed post-5 km running tests within 5 min after finishing the 5 km run; the procedures were the same as the pre-5 km tests. There was no significant difference in the self-selected speed between runner groups before and after the 5 km run (novice runners pre-5 km: 3.28 ± 0.28 m/s, post-5 km: 3.29 ± 0.32 m/s; experienced runners pre-5 km: 3.36 ± 0.33 m/s, post-5 km: 3.41 ± 0.32 m/s). The average number of attempts taken to achieve three successful trials was 4.47 ± 1.22 times. All markers were attached to runners during the entire test.

### 2.3. Data Analysis

Visual 3D software (c-motion Inc., Germantown, MD, USA) was used to process calculations of the right lower-limb joint kinematics and kinetics of the running stance phase. For the denoising process of marker trajectories, A fourth-order low-pass Butterworth filter was used to filter kinematics and ground reaction forces at frequencies of 10 Hz and 20 Hz, respectively [36]. The stance phase was determined when the vertical GRF crossed a threshold of 20 N. A Cardan X–Y–Z rotation sequence was used to calculate ankle, knee, and hip joint angles. Inverse dynamics based on the Newton–Euler approach was used to compute the lower-limb joint moments [37]. Joint moments and ground reaction forces were normalized to the body mass and body weight of each participant, respectively. Matlab version 2019b (The Math Works, Natick, MA, USA) time-normalized the kinematic and kinetic data of the running stance phase to 101 points.

The discrete values were joint range of motion (ROM), peak joint moment, peak propulsive GRF, peak braking GRF, impact peak of vertical GRF, and vertical average loading rate of GRF (VALR). The maximal value of the anterior–posterior GRF was defined as the peak propulsive GRF, while the minimal value was the peak braking GRF. The impact peak of vertical GRF was identified as the first peak of vertical GRF. VALR was the average slope calculated from 20% to 80% of the running stance phase from the initial foot contact to the impact peak of vertical GRF. These specific values have been associated with running-related injuries [17,38].

The PCA method applied in the study was based on an approach described previously [29]. For every dimension of the joint angle, moment, and GRF waveforms, the ensemble curves were separately combined into a matrix for PCA. Thus, PCA was performed on 21 separate $X_{n\times p}$ matrices, where n is the number of running trials, and p represents the 101 data points of the stance phase. For the present analysis, the waveforms of the three trials of 15 novice runners and 15 experienced runners for each of the two running conditions were inputted as row vectors, yielding $X_{180\times 101}$ matrices for each interest variable, resulting in the display of principal component models as follows:

(1) $$X_{180\times 101}=\left[\begin{array}{cccc} x_{1,1} & x_{1,2} & \cdots & x_{1,101}\\ x_{2,1} & x_{2,2} & \cdots & x_{2,101}\\ \vdots & \vdots & \ddots & \vdots \\ x_{180,1} & x_{180,2} & \cdots & x_{180,101} \end{array}\right].$$

The waveform data were transformed into uncorrelated principal components. The covariance matrix $S_{101\times 101}$ was subjected to eigenvalue analysis to perform PCA; ${\bar{x}}_{1\times 101}$ was the mean waveform of $X_{180\times 101}$ at each timepoint.

(2) $$S_{101\times 101}=\frac{\left(X_{180\times 101}-\left(1_{180\times 1}\times {\bar{x}}_{1\times 101}\right)\right)\prime \times \left(X_{180\times 101}-\left(1_{180\times 1}\times {\bar{x}}_{1\times 101}\right)\right)}{\left(180-1\right)}.$$

The eigenvector matrix $U_{101\times 101}$ was determined by orthonormalizing $S_{101\times 101}$. The columns of $U=u_{1},u_{2},\ldots u_{100}$ are named PC loading vectors. The spread along the direction of the eigenvectors was explained by the corresponding eigenvalues $L_{1\times 101}$.

(3) $$L_{1\times 101}=diag\left(\;U_{101\times 101}\prime \times S_{101\times 101}\times U_{101\times 101}\right).$$

After $U_{101\times 101}$ and $L_{1\times 101}$ determined, the PC scores $Z_{180\times 101}$ for each waveform could be computed by the deviation of each waveform trial from the overall mean with the transpose of the eigenvector matrix. Thus, each runner’s raw waveform was transformed into a set of PC scores, which indicate the similarity of their waveform shape to each specific feature.

(4) $$Z_{180\times 101}=\left(X_{180\times 101}-\left(1_{180\times 1}\times {\bar{x}}_{1\times 101}\right)\right)\times U_{101\times 101}^{\prime }.$$

To assess the adequacy of the retained principal components in representing the original data, a residual analysis was completed using the Q-statistic. The Q-statistic is computed as the sum of squared residuals between the original waveform and the reconstructed curve generated from the retained PCs [37].

In this study, the first k PCs required to be retained were determined by 90% trace criteria [28,30]. This criterion ensures that the chosen PCs capture the main patterns of variation and account for a significant portion of the overall variation in the running data. k is the number of PCs retained in the model (k≤n).

The interpretation of PCA involved visually analyzing the PC loading vectors and examining the waveforms that obtained low and high scores on each PC. This approach allowed for a comprehensive understanding of the relationships between the PCs and the corresponding waveform patterns. The high PC waveforms were defined by one standard deviation above (plus SD) each PC, and the low PC waveforms were defined by one standard deviation below (minus SD) each PC [31,39]. All the PCA processing calculations were completed in Matlab.

### 2.4. Statistical Analysis

The Shapiro–Wilk test confirmed that the PC scores and other discrete values of lower-limb biomechanics retained for analysis were normally distributed. Independent *t*-tests were employed to compare the demographics and running experience between novice and experienced runners; the significance level (alpha) was set at 0.05. A two-way repeated-measures ANOVA was conducted to quantify the main effects of running experience levels and 5 km run factors, as well as their interaction; statistical significance was accepted at α = 0.05. A Bonferroni correction adjusted post hoc pairwise comparisons to α = 0.008 when the significant interaction effect was observed [40,41]. All data were presented as means (SD). Statistical analyses were completed using SPSS 25.0 (IBM, Armonk, NY, USA).

## 3. Results

### 3.1. Discrete Variables

**Figure 2:**
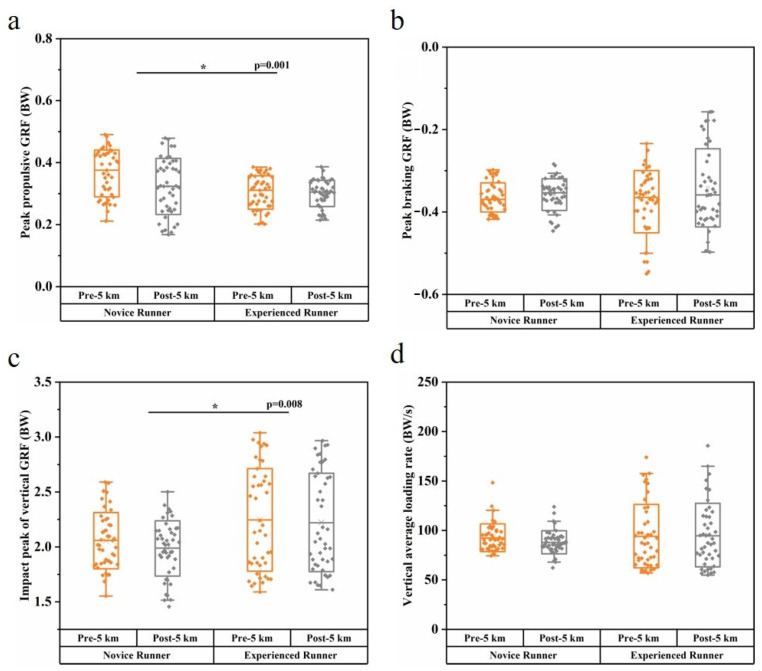
Comparison mean (SD) of GRF variables between novice and experienced runners with a 5 km run. Significant differences between runners are highlighted with an asterisk (*). (**a**) Peak propulsive GRF; (**b**) peak braking GRF; (**c**) impact peak of vertical GRF; (**d**) vertical average loading rate.

The angle ROM of the ankle Dorsi/Plant, knee Ext/Flex, and hip Adduct/Abduct were significantly different between runners with and without running experience, where novice runners showed greater ankle Invert/Evert ROM (*p* = 0.035) and hip Adduct/Abduct ROM (*p* < 0.001), but smaller knee Ext/Flex ROM (*p* < 0.001) than experienced runners (Table 2). In both novice and experienced runners, the post-5 km running resulted in significant in ROM of the knee Adduct/Abduct (*p* = 0.001) and hip Adduct/Abduct (*p* = 0.001) compared to the pre-5 km running (Table 2). The interaction between the running experience and the 5 km run had a significant effect only on the angle ROM of the knee Adduct/Abduct (*p* < 0.001) (Table 2). Novice runners also showed greater peak ankle inversion moment (*p* < 0.001), ankle rotation moment (*p* < 0.001), and hip abduction moment (*p* < 0.001), but smaller hip extension moment (*p* = 0.003) than experienced runners (Table 3). The 5 km run induced a smaller peak ankle plantarflexion moment (*p* < 0.001) and greater hip extension moment (*p* = 0.023) (Table 3). Effect sizes and 95% confidence intervals for comparison of joint range of motion (ROM) and moment are provided in Table S1. Novice runners showed greater peak propulsive GRF (*p* = 0.001) and smaller impact peak of vertical GRF (*p* = 0.008) than experienced runners (Figure 2).

### 3.2. PCA

**Figure 3:**
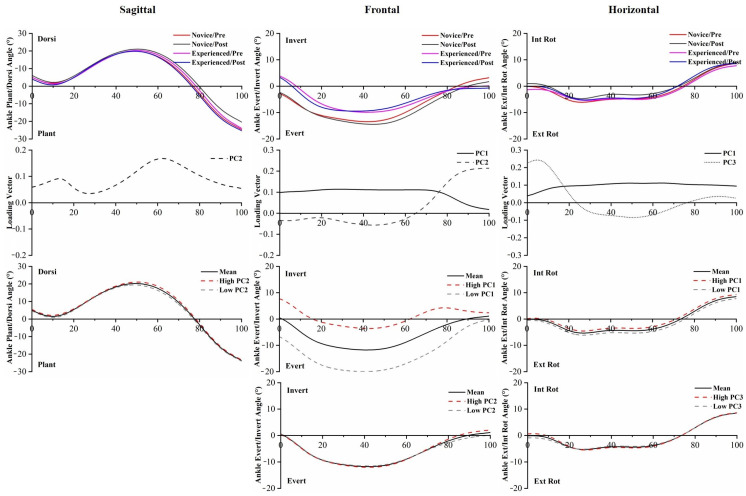
The mean of ankle angles for novice and experienced runners with a 5 km run during stance phase. The loading vectors for PC scores with significant differences. Single-component reconstruction for PC scores of three-dimension ankle angles.

The *p*-values of PC scores of joint angles are provided in Table S2. PC score statistical analysis of joint angles showed that differences were found between novice runners and experienced runners with respect to PC2 in the ankle Dorsi/Plant, and PC1 and PC2 in the ankle Invert/Evert. Statistical differences in ankle angle PC scores of the 5 km run were found in PC2 in the ankle Invert/Evert, and PC1 and PC3 in the ankle Int Rot/Ext Rot. The waveforms, PC loading vectors, and reconstructed waveforms of ankle angles are presented in Figure 3. For each variable, the waveforms were reconstructed by utilizing the scores and coefficients of the retained PCs; the high PC and low PC can be used to visually understand differences in amplitude. Experienced runners demonstrated significantly less ankle inversion angle than novice runners, which was also consistent with lower PC1 and PC2 scores than experienced runners in ankle Invert/Evert, and this magnitude difference was obvious throughout the running stance phase.

**Figure 4:**
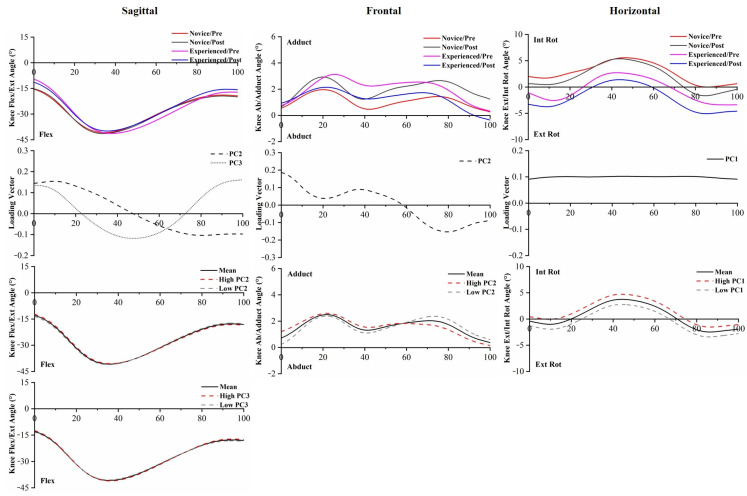
The mean of knee angles for novice and experienced runners with a 5 km run during stance phase. The loading vectors for PC scores with significant differences. Single-component reconstruction for PC scores of three-dimension knee angles.

PC score differences in knee angles between runners were found in PC2 and PC3 in the knee Ext/Flex, PC2 in the knee Adduct/Abduct, and PC1 in the knee Int Rot/Ext Rot. Statistical differences in knee angle PC scores between pre-5 km running and post-5 km running were found in PC2 in the knee Adduct/Abduct and PC1 in the knee Int Rot/Ext Rot. Compared to experienced runners, novice runners showed significantly more knee flexion angle in the early stance and more internal rotation angle throughout the running stance phase through visual inspection and PC scores. Post-5 km running showed less knee internal rotation angle than pre-5 km running (Figure 4, Table S2).

**Figure 5:**
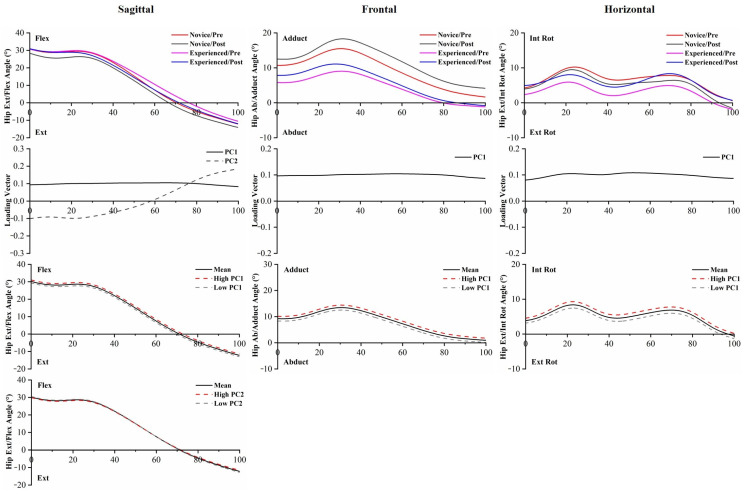
The mean of hip angles for novice and experienced runners with a 5 km run during stance phase. The loading vectors for PC scores with significant differences. Single-component reconstruction for PC scores of three-dimension hip angles.

PC score differences in hip angles between runners were found in PC1 and PC2 in the hip Flex/Ext, PC1 in the hip Adduct/Abduct, and PC1 in the hip Int Rot/Ext Rot. The effects of the 5 km run existed in PC1 in the hip Flex/Ext and PC1 in the hip Adduct/Abduct (Table S2). During the running stance phase, novice runners had significantly greater hip adduction and internal rotation angle than experienced runners. Meanwhile, post-5 km running showed a larger hip adduction angle (Figure 5). The interaction effects existed in PC3 in the knee Ext/Flex and PC1 in the hip Int Rot/Ext Rot.

**Figure 6:**
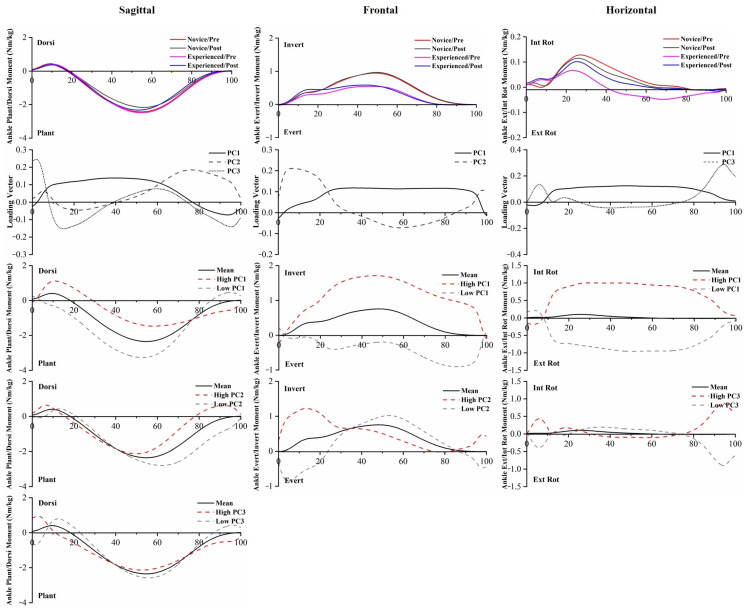
The mean of ankle moments for novice and experienced runners with a 5 km run during stance phase. The loading vectors for PC scores with significant differences. Single-component reconstruction for PC scores of three-dimension ankle moments.

The *p*-values of PC scores of joint moments are provided in Table S3. The analysis showed significant runner main effects in PC3 in the ankle Dorsi/Plant, PC1 and PC2 in the ankle Invert/Evert, and PC1 and PC3 in the ankle Int Rot/Ext Rot. The significant 5 km running main effects of ankle moments were found in PC1, PC2, and PC3 in the ankle Dorsi/Plant, and PC3 in the ankle Int Rot/Ext Rot. Compared to experienced runners, novice runners showed significantly larger ankle inversion moment and internal rotation moment throughout the stance phase. After 5 km of running, the ankle plantarflexion moment was smaller during the middle and later stances (Figure 6).

**Figure 7:**
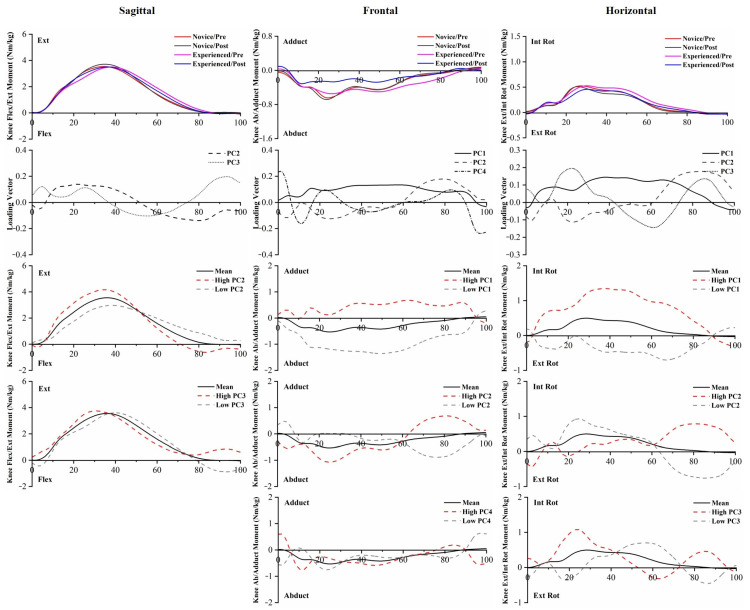
The mean of knee moments for novice and experienced runners with a 5 km run during stance phase. The loading vectors for PC scores with significant differences. Single-component reconstruction for PC scores of three-dimension knee moments.

PC score differences in knee moments were found between runners in PC2 and PC3 in the Ext/Flex, PC2 and PC4 in the Adduct/Abduct, and PC1, PC2, and PC3 in the Int Rot/Ext Rot. The significant 5 km running main effects of knee moments were found in PC2 and PC3 in the knee Ext/Flex, PC1, PC2, and PC4 in the knee Adduct/Abduct, and PC1 in the knee Int Rot/Ext Rot. Knee moment-related waveforms are presented in Figure 7.

**Figure 8:**
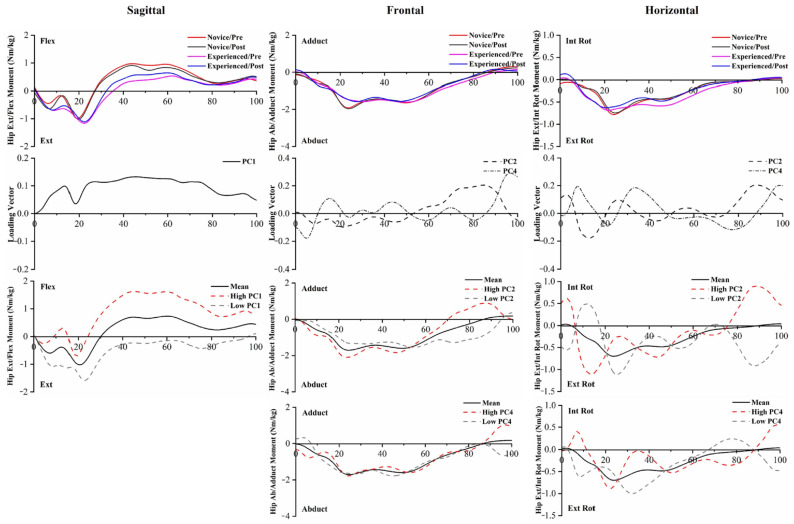
The mean of hip moments for novice and experienced runners with a 5 km run during stance phase. The loading vectors for PC scores with significant differences. Single-component reconstruction for PC scores of three-dimension hip moments.

Hip moment PC score differences between runners were found in PC1 in the hip Flex/Ext, PC2 and PC4 in the hip Adduct/Abduct, and PC2 in the hip Int Rot/Ext Rot. The significant 5 km running main effects of hip moments were found in PC2 in the hip Adduct/Abduct. Compared to experienced runners, novice runners showed significantly greater hip flexion moment throughout the running stance phase and greater external rotation moment in the early phase (Figure 8, Table S3). The interaction effects existed in PC1 in the ankle Int Rot/Ext Rot, PC1 in the knee Adduct/Abduct, and PC1 in the hip Flex/Ext.

**Figure 9:**
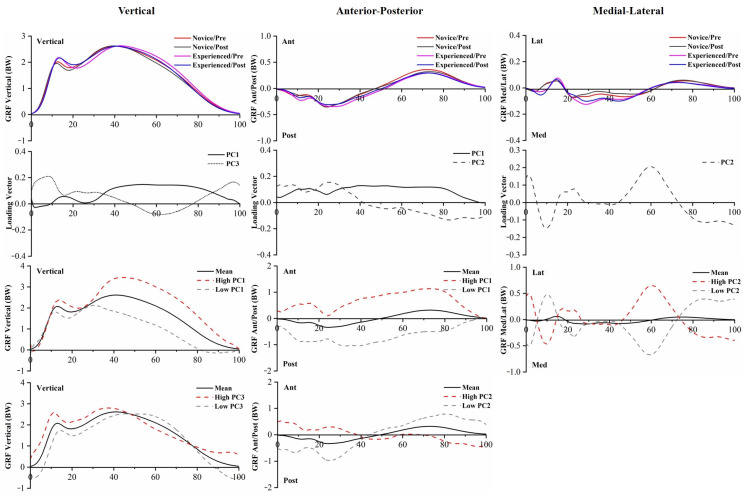
The mean of GRFs for novice and experienced runners with a 5 km run during stance phase. The loading vectors for PC scores with significant differences. Single-component reconstruction for PC scores of three-dimension GRFs.

The *p*-values of PC scores of GRFs are provided in Table S4. PC score differences of GRFs between runners were found in P1 and PC3 in vertical GRF, PC1 and PC2 in anterior–posterior GRF, and PC2 in medial–lateral GRF. PC1 in vertical GRF and PC2 in anterior-posterior GRF had significant 5 km running main effects. The raw waveforms, PC loading vectors, and reconstructed waveforms of GRFs are presented in Figure 9.

## 4. Discussion

The purpose of this study was to analyze the biomechanical effects of a 5 km run between novice and experienced runners. Differences in lower-limb kinematics and kinetics during a prolonged running session between novice runners and experienced runners were found. For the discrete variables obtained by a two-way repeated-measures ANOVA, the joint ROM and peak joint moment showed differences between novice runners and experienced runners. The peak propulsive GRF was greater in novice runners, while the impact peak of vertical GRF in novice runners was smaller. For the PC modeling of waveforms, it was observed that the first four PCs accounted for the most variations, ranging from 86.52% to 96.16% for all biomechanical variables investigated, which is consistent with the literature [24,27,39]. PCs were a set of orthogonal waveform features obtained after principal component analysis of mixed biomechanical waveforms from multiple subjects. Typically, four PCs can be used to explain the main variation in a dataset. Using a PCA approach may offer unique insights into the underlying patterns of running biomechanical waveforms. These findings partially supported our hypothesis.

### 4.1. Kinematics

The runner’s experience was expected to influence running performance and injury risks by altering lower-extremity kinematics and kinetics. Consistent with this assumption, novice runners showed greater ankle Invert/Evert ROM, which was believed to be associated with running-related injuries [42]. Meanwhile, experienced runners had greater knee Ext/Flex ROM; this finding is in agreement with previous research [23]. The greater knee flexion ROM appears to be a protective adaptation in experienced runners, as previous studies have suggested that a greater knee flexion angle during stance can reduce the ground reaction force and attenuate shock impacts above the knee joint [43]. Our results showed that novice runners had greater hip Adduct/Abduct ROM; increased hip adduction has been identified as a potential risk factor for common running injuries such as iliotibial band syndrome [14]. After a 5 km run, the ROM of the knee adduct/abduct and hip adduct/abduct increased. The accumulated fatigue of hip abductor muscle-tendon units (tensor fasciae latae, gluteus medius, and gluteus minimus) may be causing the increase in hip adduction, and hip musculature is essential in overcoming substantial external hip adduction moments [44]. The stability of the hip joint may prevent running-related injuries to a certain extent. Similarly, Willwacher et al. [45] found clear changes in Adduct/Abduct and Int Rot/Ext Rot joint kinematics after a 10 km long-distance run.

Even though no statistical difference exists in standard discrete value analysis of the lower limb, PCA was capable of recognizing significant differences in the waveforms of joint angles between novice and experienced runners with the prolonged running session. PC1 captured the general magnitude differences in the data, PC2 primarily captured the differences in timing, and PC3 extracted differences in relative amplitudes [31]. PC1 and PC2 of the ankle Invert/Evert angle captured the significantly greater eversion angle in novice runners with respect to experienced runners, similar to the findings reported by Maas et al. [23]. The high eversion angle of the ankle joint has been linked to a higher risk of injury development in runners. It has been hypothesized that increased ankle eversion can lead to greater medial foot displacement, which is associated with increased tibial abduction [15,46]. Novice runners should be mindful of changes occurring in the ankle joint during running, particularly the eversion angle, and make necessary adjustments promptly. PC2 captured subtle shifts in the timing of the peak knee flexion angle, while PC3 reflected an increase in the knee flexion angle during early stance, specifically among novice runners. In hip Flex/Ext, PC2 and PC3 revealed that experienced runners exhibited a greater hip flexion angle than novice runners, which is consistent with a previous study [12]. The increased knee internal rotation angle, increased hip adduction angle, and increased hip internal rotation angle [47,48,49] have been associated with running-related injuries, especially iliotibial band syndrome, which were reflected in PC1 of the knee Int Rot/Ext Rot, hip Adduct/Abduct, and hip Int Rot/Ext Rot among novice runners. Compensatory femoral internal rotation caused by excessive tibial internal rotation during stance may lead to knee stress injuries [46,50]. These kinematic changes in novice runners may indicate a lack of control over running technique, while experienced runners may exhibit greater control.

### 4.2. Kinetics

There were also significant differences between the experience levels of runners for the kinetic variables. The peak ankle inversion moment and peak internal rotation moment of novice runners were greater than those of experienced runners. An increase in ankle inversion moment indicates that novice runners may have increased demands on the ankle varus muscles, including the anterior tibialis and posterior tibialis, which play a role in eccentrically supporting the plantar arch during the stance phase [33]. At the hip joint, the extension moment of novice runners was reduced, and the abduction moment was increased. The lack of running experience may be related to an imbalance of hip muscles. Meanwhile, GRF variable comparisons across experience levels revealed significant differences in peak propulsive GRF and vertical impact peak. To maintain stability and forward propulsion during the second half of the stance phase, the body’s propulsive ground reaction force (GRF) has to increase in proportion to the braking GRF [18,46]. Therefore, the increased peak propulsive GRF of novice runners indicates that this may be an adaptation to stabilize running posture. In our study, we noticed the impact peak of vertical GRF was different between runners. However, some researchers did not find any differences in impact peak between runners with different running experiences [10,14], and the influence of the impact peak of vertical GRF on running-related injuries remains controversial [14]. The decreases in ankle plantarflexion moment and knee extension moment were noticed during post-5 km running. These changes in biomechanics after a prolonged running session were consistent with previous research [47].

PC1 and PC2 of the ankle Invert/Evert moment captured the differences in magnitude and amplitude between the two groups, reporting a significantly greater inversion moment in novice runners compared to experienced runners throughout the entire stance phase. PC1 and PC3 of the ankle Int Rot/Ext Rot moment captured the differences between experienced runners and novice runners, showing that novice runners have a greater ankle internal rotation moment than experienced runners. The greater moment can reflect an increase in antagonistic activity and, thus, may indicate increased joint load. PC2 and PC3 extracted phase shift and amplitude differences in the knee Ext/Flex moment, while PC2 extracted phase shift differences in the knee Adduct/Abduct moment. This time delay would decrease the loading rate during the initial stance to midstance, which has been considered a risk factor for overuse running injuries. Given the relatively modest variance explained, it was difficult to distinguish the influence expressed by PC4. The increased knee internal rotation moment throughout the entire stance phase may be an unintended effect of running, as it has been linked to the progression of knee osteoarthritis during gait [51]. In the hip joint, PC1 captured the magnitude difference in the flexion moment, which was consistent with the hip Flex/Ext ROM.

Running GRFs are associated with impact shock, loading accumulation, and stress syndrome in the lower limb [39]. While comparing the PC scores between novice and experienced runners, the vertical GRF varied in the PC1 of magnitude variances and the PC3 of relative amplitudes; however, the comparison of the VALR showed no significant difference between runners. Experienced runners showed greater vertical and posterior GRFs than novice runners. Increases in GRF parameters may not be a direct signal of tibia bone stresses or overuse injury risks, as these may be associated with other intrinsic muscle contributions and mechanical alignment [19]. Meanwhile, kinetic differences between pre-5 km running and post-5 km running were reflected in PC scores of joint moments, especially in the ankle and knee joints. The reduced plantarflexion moment may be due to the decrease in energy absorption caused by sustained running, and the decreased knee extension moment during the middle stance and later stance may indicate that runners have weak extensor muscles after a 5 km run. Although no significant differences were found between pre-5 km and post-5 km running for the discrete variables of GRFs, PCA analysis was still crucial for identifying the main effects over the course of the run. PC1 of the vertical GRF and PC2 of the anterior–posterior GRF indicate a systematic alteration in running GRFs over the course of the run, which could be explained by a certain degree of fatigue in the lower-extremity musculature.

While the traditional two-way repeated-measures ANOVA can only perform statistical analysis on discrete values, PCA can perform dimensionality reduction analysis on the entire time series curve. PCA captured differences in the magnitude and amplitude of lower-extremity biomechanical waveforms by retaining at least 90% of the available information [30]. Using single-component reconstruction, the lower-limb joint angles, joint moments, and GRFs collected by PCA can be interpreted visually. In fact, this method may offer a robust and clinically relevant interpretation. In the current study, PCs generated from lower-extremity kinematics and kinetics were shown to be indicators of running experience effects and prolonged running effects. Results from our study suggest that running experience may influences the running mechanics of runners, especially those commonly associated with running-related injuries. Biomechanical changes during post-5 km running might be associated with a fatigued state and may help to understand potential alterations due to overuse injuries [52,53].

### 4.3. Limitations

Several limitations in this study should be acknowledged. Firstly, the running biomechanics differences in our study may have been affected by running speed, as we collected gait data at the preferred running speed of runners rather than a uniform speed to ensure a more natural gait pattern. Moreover, it is important to note that the speed recorded in this study represents the average speed between the two timing gates positioned 2 m apart, rather than using a treadmill to maintain a consistent speed, which may have caused errors in the speed calculations. Abbasi et al. [54] suggested that gait coupling patterns changed as running speed varied. Orendurff et al. [55] found that running speed affects lower-limb joint biomechanics, especially in maximal kinematic and kinetic variables of the hip, knee, and ankle joints. However, a few studies [56] indicated that running speed does not have a significant influence on the lower-limb biomechanical asymmetry of runners. In order to gain a better understanding of this aspect, our future research will focus on determining the influence of various running speeds on lower-limb biomechanics.

Secondly, we investigated how a prolonged running session influences gait data, thus using a 5 km run protocol rather than a fatigue run protocol. Different runners have different reactions to the 5 km run; most novice runners have reached an exerted fatigue state after a 5 km run, while experienced runners have not. Thirdly, due to limitations in laboratory and experimental equipment, we conducted our data collection overground, whereas the 5 km running was performed on a treadmill. It is important to note that running on different surfaces can potentially introduce biomechanical differences to some extent, which should be avoided in future research to ensure more accurate and consistent findings [57,58]. Furthermore, we investigated only male runners; as gender differences exist in running biomechanics, our findings may not apply to female runners. Future studies could perform this kind of analysis on female runners. Another limitation is that muscle activities were not included in the present study. Muscle activities can differ across experience levels and running states, and it can provide unique insights into gait analysis. We also did not explore the influence of muscle fibers on running biomechanics, which may have implications for running technique. These limitations should be considered in future studies.

## 5. Conclusions

Running mechanics differences between novice and experienced runners were assessed for a 5 km run using traditional discrete variables and PCA with single-component reconstruction for waveform analysis. The results of this study suggest that the PCA approach can provide unique insight into running biomechanics and injury mechanisms. The findings from this study showed that running experience had an impact on lower-limb biomechanics. Novice runners exhibited greater changes in joint angles, joint moments, and GRFs than experienced runners, and those patterns may relate to lower-limb injuries, which could potentially guide training program developments and injury prevention protocols for runners with different running experiences. Furthermore, future work could prospectively investigate runners with different running experiences to further understand whether certain biomechanical variables can be used to improve running performance and identify injury risks.

## Figures and Tables

**Table 1:** Mean (SD) of participant characteristics of novice and experienced runners.

Variable	Novice	Experienced	*p*-Value
Age (years)	23.80 (1.97)	23.65 (1.67)	0.398
Height (m)	1.76 (0.49)	1.75 (0.56)	0.702
Weight (kg)	71.93 (7.70)	72.73 (6.44)	0.794
BMI (kg/m^2^)	23.13 (1.18)	23.65 (1.67)	0.456
Running experience (years)	1.53 (0.74)	6.07 (1.62)	<0.001
Running volume (km/week)	7.13 (2.67)	38.33 (7.72)	<0.001

Note: Significant difference (*p* < 0.05).

**Table 2:** Mean (SD) of joint range of motion (ROM) for novice and experienced runners of pre-5 km run and post-5 km run.

Joint	ROM (°)	Novice/Pre	Novice/Post	Experienced/Pre	Experienced/Post	Runner	5 km	Interaction
						Main Effect	Main Effect	Effect
Ankle	Dorsi/Plant	44.80 (10.77)	41.71 (6.41)	44.55 (6.41)	45.41 (7.96)	F = 0.878; *p* = 0.355	F = 2.270; *p* = 0.139	F = 3.515; *p* = 0.078
	Invert/Evert	17.16 (4.82)	17.21 (4.75)	15.32 (2.91)	16.04 (1.91)	F = 4.720; *p* = 0.035	F = 1.442; *p* = 0.236	F = 1.104; *p* = 0.299
	Int Rot/Ext Rot	14.94 (2.97)	14.20 (1.50)	13.77 (2.41)	14.07 (2.84)	F = 1.978; *p* = 0.167	F = 0.720; *p* = 0.401	F = 4.024; *p* = 0.051
Knee	Ext/Flex	26.18 (4.05)	27.14 (3.26)	32.23 (3.55)	29.90 (2.94)	F = 57.932; *p* < 0.001	F = 1.941; *p* = 0.171	F = 5.917; *p* = 0.035
	Adduct/Abduct	2.85 (0.63)	3.90 (1.55)	3.38 (0.79)	3.43 (1.20)	F = 0.025; *p* = 0.876	F = 12.818; *p* = 0.001	F = 21.117; *p* < 0.001
	Int Rot/Ext Rot	6.62 (2.28)	6.70 (1.98)	7.73 (2.67)	7.73 (2.40)	F = 0.033; *p* = 0.857	F = 4.675; *p* = 0.057	F = 2.572; *p* = 0.090
Hip	Flex/Ext	43.17 (3.12)	42.81 (3.05)	41.98 (3.91)	43.12 (5.41)	F = 0.503; *p* = 0.482	F = 0.676; *p* = 0.415	F = 5.406; *p* = 0.025
	Adduct/Abduct	14.10 (3.66)	14.76 (4.68)	10.37 (1.90)	12.00 (1.22)	F = 23.459; *p* < 0.001	F = 13.369; *p* = 0.001	F = 2.967; *p* = 0.092
	Int Rot/Ext Rot	10.96 (4.44)	12.66 (6.26)	10.48 (3.31)	10.69 (2.61)	F = 1.378; *p* = 0.247	F = 6.664; *p* = 0.013	F = 5.682; *p* = 0.022

Note: Dorsi/Plant = dorsiflexion/plantarflexion, Invert/Evert = inversion/eversion, Int Rot/Ext Rot = internal rotation/external rotation, Ext/Flex = extension/flexion, Adduct/Abduct = adduction/abduction, Flex/Ext = flexion/extension. Significant difference (*p* < 0.05). The significant differences in interaction effect were determined using Bonferroni corrections (α = 0.008).

**Table 3:** Mean (SD) of peak joint moment of the stance phase for novice and experienced runners of pre-5 km run and post-5 km run.

Joint	Moment (Nm/kg)	Novice/Pre	Novice/Post	Experienced/Pre	Experienced/Post	Runner	5 km	Interaction
						Main Effect	Main Effect	Effect
Ankle	Plantarflexion	2.46 (0.43)	2.20 (0.39)	2.50 (0.29)	2.33 (0.16)	F = 1.864; *p* = 0.179;	F = 76.958; *p* < 0.001	F = 4.376; *p* = 0.042
	Inversion	0.95 (0.23)	0.98 (0.27)	0.56 (0.27)	0.73 (0.16)	F = 78.585; *p* < 0.001	F = 2.511; *p* = 0.051	F = 3.050; *p* = 0.088
	Internal rotation	0.16 (0.09)	0.16 (0.10)	0.09 (0.07)	0.12 (0.07)	F = 17.016; *p* < 0.001	F = 3.297; *p* = 0.076	F = 4.370; *p* = 0.042
Knee	Extension	3.56 (0.33)	3.74 (0.39)	3.53 (0.47)	3.53 (0.48)	F = 1.366; *p* = 0.249	F = 2.589; *p* = 0.079	F = 5.268; *p* = 0.027
	Abduction	0.67 (0.24)	0.74 (0.21)	0.70 (0.11)	0.58 (0.17)	F = 3.770; *p* = 0.059	F = 1.825; *p* = 0.184	F = 4.638; *p* = 0.041
	Internal rotation	0.54 (0.06)	0.52 (0.15)	0.56 (0.12)	0.50 (0.08)	F = 0.001; *p* = 0.991	F = 8.547; *p* = 0.005	F = 2.263; *p* = 0.140
Hip	Extension	1.08 (0.26)	1.26 (0.32)	1.37 (0.40)	1.39 (0.33)	F = 9.957; *p* = 0.003	F = 5.519; *p* = 0.023	F = 5.721; *p* = 0.083
	Abduction	1.98 (0.32)	2.05 (0.43)	1.77 (0.25)	1.79 (0.25)	F = 16.390; *p* < 0.001	F = 1.890; *p* = 0.176	F = 0.933; *p* = 0.339
	External rotation	0.83 (0.23)	0.82 (0.27)	0.87 (0.15)	0.80 (0.20)	F = 0.138; *p* = 0.712	F = 3.352; *p* = 0.074	F = 2.228; *p* = 0.143

Note: Significant difference (*p* < 0.05). The significant differences in interaction effect were determined using Bonferroni corrections (α = 0.008).

## Data Availability

The data that support the findings of this study are available on reasonable request from the corresponding author. The data are not publicly available due to privacy or ethical restrictions.

## Supplementary Materials

The following supporting information can be downloaded at https://www.mdpi.com/article/10.3390/bioengineering10070876/s1, Table S1. Effect sizes and 95% confidence intervals for comparison of joint range of motion (ROM) and moment; Table S2. Mean (SD) of joint angles for all principal components (PCs) retained according to the 90% trace criterion; Table S3. Mean (SD) of joint moments for all principal components (PCs) retained according to the 90% trace criterion; Table S4. Mean (SD) of ground reaction forces (GRFs) for all principal components (PCs) retained according to the 90% trace criterion.
