# Supplementary material for: PCA of Running Biomechanics after 5 km between Novice and Experienced Runners
Source: Bioengineering (Basel). 2023 Jul 24;10(7):876. doi: 10.3390/bioengineering10070876 (PMC10376576; doi:10.3390/bioengineering10070876)
Supplement: Supplementary file 1 [file bioengineering-10-00876-s001.zip › bioengineering-2460608-supplementary.pdf]

Table S1. Effect sizes and 95% confidence intervals for comparison of joint range of motion (ROM) and moment.

Variable		Novice pre vs. post		Experienced pre vs. post		Novice pre vs. Experienced pre		Novice post vs Experienced post	
		Cohen's d	95% CI	Cohen's d	95% CI	Cohen's d	95% CI	Cohen's d	95% CI
Joint ROM									
Ankle	Dorsi/Plant	0.35	40.53–45.98	0.12	43.05–46.91	0.03	43.03–46.32	0.51	41.94–45.19
	Invert/Evert	0.01	15.78–18.60	0.29	15.23–16.14	0.46	15.23–17.26	0.32	15.99–17.26
	Int Rot/Ext Rot	0.31	14.01–15.13	0.11	13.19–14.65	0.43	13.78–14.93	0.06	13.66–14.61
Knee	Ext/Flex	0.26	25.81–27.51	0.71	30.26–31.87	1.59	28.33–30.08	0.89	27.87–29.17
	Adduct/Abduct	0.89	3.10–3.65	0.05	3.14–3.67	0.74	2.97–3.26	0.34	3.36–3.97
	Int Rot/Ext Rot	0.04	7.18–8.14	0	7.08–8.38	0.45	6.73–7.62	0.47	7.70–8.72
Hip	Flex/Ext	0.12	42.21–43.77	0.24	41.28–43.82	0.34	41.79–43.37	0.07	41.84–44.08
	Adduct/Abduct	0.16	13.27–15.59	1.02	10.84–11.53	1.28	11.67–12.80	0.81	12.72–14.04
	Int Rot/Ext Rot	0.31	10.29–13.33	0.07	9.76–11.41	0.12	10.12–11.32	0.41	10.84–12.51
Joint Moment									
Ankle	Plantarflexion	0.63	2.21–2.45	0.73	2.35–2.48	0.11	2.39–2.57	0.44	2.21–2.33
	Inversion	0.12	0.93–1.01	0.77	0.59–0.71	1.56	0.71–0.81	1.13	0.81–0.90
	Internal rotation	0	0.13–0.19	0.43	0.09–0.13	0.87	0.11–0.15	0.46	0.12–0.16
Knee	Extension	0.50	3.55–3.74	0	3.66–3.40	0.07	3.47–3.61	0.49	3.57–3.69
	Abduction	0.31	0.65–0.76	0.84	0.61–0.67	0.16	0.65–0.72	0.84	0.62–0.70
	Internal rotation	0.18	0.50–0.56	0.59	0.50–0.55	0.21	0.53–0.57	0.17	0.48–0.53
Hip	Extension	0.62	1.10–1.24	0.05	1.28–1.48	0.86	1.15–1.30	0.40	1.27–1.38
	Abduction	0.18	1.92–2.11	0.08	1.71–1.85	0.73	1.81–1.93	0.74	1.84–2.00
	External rotation	0.04	0.76–0.89	0.40	0.79–0.88	0.21	0.80–0.90	0.08	0.76–0.86

Note: CI = confidence interval.

Table S2. Mean (SD) of joint angles for all principal components (PCs) retained according to the 90% trace criterion.

Joint	Angle	PC	Variance Explained (%)	Mean (SD) PC scores				Runner p-Value	5 km p-Value	Interaction p-Value
				Novice/Pre	Novice/Post	Experienced/Pre	Experienced/Post	Main Effect	Main Effect	Effect
Ankle	Dorsi/Plant	1	47.67	0.77 (9.57)	−0.68 (7.34)	−0.88 (3.05)	0.79 (6.21)	0.950	0.868	0.012
		2	32.94	−1.23 (5.46)	3.67 (6.02)	0.29 (4.63)	−2.72 (4.99)	0.030	0.192	0.025
		3	13.91	0.69 (1.76)	−0.42 (1.90)	−0.16 (4.61)	−0.11 (5.34)	0.674	0.176	0.177
	Invert/Evert	1	73.62	−2.86 (7.75)	−5.02 (7.66)	3.90 (7.92)	3.98 (7.15)	<0.001	0.117	0.100
		2	20.65	2.12 (3.47)	0.97 (3.18)	−1.43 (4.04)	−1.67 (5.99)	<0.001	0.014	0.100
	Int Rot/Ext Rot	1	64.56	−2.25 (6.78)	3.92 (7.02)	−2.85 (8.60)	1.18 (8.09)	0.284	<0.001	0.206
		2	14.51	1.34 (4.08)	−1.48 (3.92)	−0.79 (3.43)	0.93 (3.20)	0.845	0.126	0.011
		3	11.04	−0.18 (3.33)	0.46 (4.61)	−0.87 (2.85)	0.60 (1.87)	0.705	0.007	0.217
Knee	Ext/Flex	1	66.67	−0.53 (8.81)	−1.41 (9.54)	−0.75 (8.67)	2.69 (4.44)	0.213	0.209	0.023
		2	19.17	−1.98 (4.42)	−1.42 (3.96)	3.07 (4.44)	0.33 (3.26)	<0.001	0.020	0.010
		3	9.81	−2.12 (3.04)	−1.63 (3.07)	2.73 (1.32)	1.01 (1.97)	<0.001	0.093	<0.001
	Adduct/Abduct	1	85.74	−2.72 (8.00)	2.63 (12.66)	1.98 (4.49)	−1.89 (9.25)	0.964	0.352	0.017
		2	6.67	0.07 (1.64)	−2.09 (2.87)	0.25 (2.50)	1.76 (1.59)	<0.001	0.148	0.021
	Int Rot/Ext Rot	1	90.20	4.95 (5.79)	2.98 (7.02)	−2.30 (5.31)	−5.64 (13.69)	<0.001	0.018	0.416
Hip	Flex/Ext	1	83.65	0.70 (6.10)	−5.84 (11.02)	4.89 (5.88)	0.24 (9.29)	0.007	<0.001	0.327
		2	10.23	−0.98 (2.27)	−0.46 (2.63)	1.26 (3.04)	0.18 (4.22)	0.003	0.452	0.040
	Adduct/Abduct	1	86.23	14.10 (3.66)	14.76 (4.68)	10.37 (1.90)	12.00 (1.22)	<0.001	0.001	0.092
		2	9.29	−0.56 (3.25)	0.29 (4.56)	0.85 (1.70)	−0.58 (1.55)	0.655	0.372	0.028
	Int Rot/Ext Rot	1	75.65	2.85 (8.50)	0.32 (9.08)	−4.22 (6.00)	1.05 (9.60)	0.035	0.095	0.001
		2	16.76	−0.13 (4.20)	−0.96 (5.16)	0.08 (3.57)	1.01 (3.14)	0.256	0.829	0.023

Note: Dorsi/Plant = dorsiflexion/plantarflexion, Invert/Evert = inversion/eversion, Int Rot/Ext Rot = internal rotation/external rotation, Ext/Flex = extension/flexion, Adduct/Abduct = adduction/abduction, Flex/Ext = flexion/extension. Significant difference ($p < 0.05$). The significant differences in interaction effect were determined using Bonferroni corrections ($\alpha = 0.008$).

Table S3. Mean (SD) of joint moments for all principal components (PCs) retained according to the 90% trace criterion.

Joint	Moment	PC	Variance Explained (%)	Mean (SD) PC scores				Runner p-Value	5 km p-Value	Interaction p-Value
				Novice/Pre	Novice/Post	Experienced/Pre	Experienced/Post	Main Effect	Main Effect	Effect
Ankle	Dorsi/Plant	1	50.03	−1.25 (9.46)	2.46 (7.90)	−0.75 (5.82)	−0.46 (3.34)	0.369	0.001	0.013
		2	27.92	−1.24 (4.74)	1.19 (6.02)	−2.81 (3.74)	2.85 (4.76)	0.966	<0.001	0.020
		3	10.50	1.04 (2.40)	0.09 (2.65)	−0.29 (3.70)	−0.84 (3.85)	0.017	<0.001	0.576
		4	4.96	1.02 (2.23)	−0.63 (2.13)	0.54 (2.23)	−0.93 (1.81)	0.334	0.051	0.632
	Invert/Evert	1	67.39	3.85 (7.53)	4.40 (7.42)	−5.04 (7.05)	−3.21 (6.63)	<0.001	0.197	0.579
		2	19.20	−1.09 (3.90)	−0.83 (4.87)	−0.87 (2.96)	2.79 (4.53)	<0.001	0.072	0.025
		3	6.21	0.20 (3.79)	0.08 (2.27)	0.03 (1.38)	−0.32 (2.00)	0.481	0.483	0.717
	Int Rot/Ext Rot	1	62.80	2.14 (7.29)	1.10 (8.74)	−3.48 (7.58)	0.24 (6.66)	0.007	0.062	0.001
		2	15.69	0.56 (3.53)	0.53 (2.87)	−1.41 (3.96)	0.31 (4.13)	0.143	0.067	0.011
		3	9.59	0.57 (3.62)	0.71 (2.41)	−1.73 (2.82)	0.44 (2.76)	0.007	0.001	0.016
		4	5.54	−0.36 (1.86)	0.01 (2.33)	0.10 (1.89)	0.25 (3.18)	0.254	0.313	0.671
Knee	Ext/Flex	1	37.17	−1.72 (4.67)	−0.98 (5.05)	2.59 (7.33)	0.11 (6.38)	0.053	0.091	0.062
		2	26.09	1.42 (5.23)	2.04 (3.86)	−3.39 (5.19)	−0.07 (4.46)	0.001	<0.001	0.013
		3	19.17	1.30 (3.86)	2.20 (2.01)	−2.53 (5.13)	−0.97 (4.39)	<0.001	0.004	0.345
		4	7.62	0.60 (2.16)	−1.63 (2.79)	0.44 (2.99)	0.59 (2.50)	0.058	0.072	0.031
	Adduct/Abduct	1	49.62	−0.67 (7.60)	−0.36 (6.36)	−4.08 (5.36)	5.11 (5.72)	0.459	<0.001	<0.001
		2	18.60	1.92 (3.32)	2.49 (4.00)	−3.16 (3.81)	−1.25 (3.60)	<0.001	<0.001	0.112
		3	10.16	0.34 (1.99)	−0.18 (2.52)	−0.81 (3.88)	0.65 (3.89)	0.768	0.174	0.011
		4	8.14	−1.11 (2.61)	−0.29 (1.69)	0.16 (1.95)	1.24 (2.97)	0.001	<0.001	0.652
		5	5.52	−0.33 (2.28)	0.15 (1.68)	−0.19 (2.01)	0.37 (2.32)	0.646	0.064	0.861
	Int Rot/Ext Rot	1	48.08	−0.98 (2.93)	−3.78 (4.97)	5.29 (7.05)	−0.53 (5.83)	<0.001	<0.001	0.023
		2	18.42	−1.88 (4.30)	−1.67 (4.51)	3.08 (3.69)	0.47 (2.63)	<0.001	0.053	0.015
		3	13.18	1.01 (2.10)	1.69 (2.96)	−0.78 (4.03)	−1.92 (2.85)	<0.001	0.387	0.009
		4	11.54	0.84 (2.47)	0.55 (3.44)	0.27 (2.60)	−1.66 (3.25)	0.067	0.050	0.013

Hip	Flex/Ext	1	51.64	4.60 (4.86)	3.17 (6.92)	−5.11 (6.07)	−2.66 (5.07)	<0.001	0.457	<0.001
		2	19.41	−1.44 (5.40)	−0.07 (3.22)	0.89 (3.34)	0.62 (3.67)	0.058	0.279	0.050
		3	11.79	0.48 (2.28)	−0.63 (2.62)	0.40 (2.80)	−0.25 (3.35)	0.764	0.050	0.546
		4	6.33	−0.51 (1.83)	−0.17 (2.50)	0.27 (2.10)	0.41 (3.39)	0.082	0.439	0.639
	Adduct/Abduct	1	45.07	−0.66 (5.94)	−0.27 (8.47)	−1.35 (3.40)	2.27 (4.56)	0.393	0.051	0.024
		2	22.64	1.24 (4.06)	1.24 (4.16)	−3.79 (4.87)	1.31 (1.89)	0.003	<0.001	0.031
		3	15.04	0.19 (1.93)	0.97 (3.29)	−0.28 (4.29)	−0.89 (5.19)	0.055	0.775	0.010
		4	8.14	1.94 (2.62)	0.93 (2.61)	−1.39 (2.07)	−1.48 (2.56)	<0.001	0.059	0.150
	Int Rot/Ext Rot	1	53.41	0.95 (8.29)	1.52 (7.21)	−3.48 (6.36)	1.00 (5.41)	0.056	0.085	0.009
		2	23.35	−1.72 (3.28)	−1.50 (3.25)	0.77 (4.21)	2.45 (5.33)	<0.001	0.057	0.028
		3	7.92	−0.79 (2.17)	−0.42 (2.72)	0.74 (1.78)	0.46 (3.96)	0.051	0.898	0.229
		4	6.50	−0.81 (1.61)	−0.47 (3.64)	0.24 (2.02)	1.04 (2.18)	0.012	0.081	0.379

Note: Dorsi/Plant = dorsiflexion/plantarflexion, Invert/Evert = inversion/eversion, Int Rot/Ext Rot = internal rotation/external rotation, Ext/Flex = extension/flexion, Adduct/Abduct = adduction/abduction, Flex/Ext = flexion/extension. Significant difference ($p < 0.05$). The significant differences in interaction effect were determined using Bonferroni corrections ($\alpha = 0.008$).

Table S4. Mean (SD) of ground reaction forces (GRFs) for all principal components (PCs) retained according to the 90% trace criterion.

GRF	PC	Variance Explained (%)	Mean (SD) PC scores				Runner p-Value	5 km p-Value	Interaction p-Value
			Novice/Pre	Novice/Post	Experienced/Pre	Experienced/Post	Main Effect	Main Effect	Effect
Vertical	1	39.39	−0.15 (4.82)	−2.63 (7.45)	3.24 (5.87)	−0.47 (5.74)	0.007	<0.001	0.265
	2	26.25	−0.02 (3.97)	0.78 (4.04)	−2.79 (5.74)	2.03 (5.48)	0.499	0.082	0.025
	3	13.57	0.70 (3.29)	1.11 (2.64)	−0.68 (4.37)	−1.13 (3.91)	0.001	0.966	0.368
	4	7.85	−0.62 (2.54)	−1.87 (2.90)	1.31 (2.30)	1.17 (2.23)	0.053	0.026	0.096
	5	5.27	−0.16 (2.67)	0.41 (2.05)	−0.26 (1.98)	−0.01 (5.99)	0.470	0.156	0.600
Anterior–posterior	1	47.61	3.32 (6.04)	1.64 (5.94)	−3.71 (7.41)	−1.25 (6.27)	<0.001	0.728	0.015
	2	19.77	−2.25 (2.77)	−0.11 (4.11)	−0.20 (3.37)	2.55 (4.50)	<0.001	<0.001	0.544
	3	15.89	−0.26 (3.75)	0.66 (3.89)	0.44 (4.16)	−0.84 (4.17)	0.653	0.694	0.028
	4	8.33	−0.33 (3.14)	−0.95 (3.66)	1.09 (2.17)	0.19 (1.99)	0.058	0.090	0.645
Medial–lateral	1	66.12	1.15 (9.65)	2.01 (9.71)	−1.46 (6.52)	−1.71 (5.57)	0.053	0.663	0.353
	2	13.23	−1.51 (3.30)	−1.13 (3.49)	1.05 (2.20)	1.59 (2.62)	<0.001	0.089	0.759
	3	7.29	0.36 (1.83)	0.49 (2.07)	−0.23 (2.90)	−0.62 (3.62)	0.117	0.583	0.335
	4	4.68	−0.27 (1.79)	−0.59 (2.67)	0.62 (2.20)	0.25 (1.78)	0.050	0.141	0.935

Note: The significant differences in interaction effect were determined using Bonferroni corrections ($\alpha = 0.008$).
